# Mitochondria-Targeted Antioxidant SkQ1 Prevents Anesthesia-Induced Dry Eye Syndrome

**DOI:** 10.1155/2017/9281519

**Published:** 2017-10-12

**Authors:** Evgeni Yu. Zernii, Olga S. Gancharova, Viktoriia E. Baksheeva, Marina O. Golovastova, Ekaterina I. Kabanova, Marina S. Savchenko, Veronika V. Tiulina, Larisa F. Sotnikova, Andrey A. Zamyatnin, Pavel P. Philippov, Ivan I. Senin

**Affiliations:** ^1^Department of Cell Signaling, Belozersky Institute of Physico-Chemical Biology, Lomonosov Moscow State University, Moscow 119992, Russia; ^2^Department of Biology and Pathology of Domestic, Laboratory and Exotic Animals, Skryabin Moscow State Academy of Veterinary Medicine and Biotechnology, Moscow 109472, Russia; ^3^Institute of Molecular Medicine, Sechenov First Moscow State Medical University, Moscow 119991, Russia

## Abstract

Dry eye syndrome (DES) is an age-related condition increasingly detected in younger people of risk groups, including patients who underwent ocular surgery or long-term general anesthesia. Being a multifactorial disease, it is characterized by oxidative stress in the cornea and commonly complicated by ocular surface inflammation. Polyetiologic DES is responsive to SkQ1, a mitochondria-targeted antioxidant suppressing age-related changes in the ocular tissues. Here, we demonstrate safety and efficacy of topical administration of SkQ1 at a dosage of 7.5 *μ*M for the prevention of general anesthesia-induced DES in rabbits. The protective action of SkQ1 improves clinical state of the ocular surface by inhibiting apoptotic and prenecrotic changes in the corneal epithelium. The underlying mechanism involves the suppression of the oxidative stress supported by the stimulation of intrinsic antioxidant activity and the activity of antioxidant enzymes, foremost glutathione peroxidase and glutathione reductase, in the cornea. Furthermore, SkQ1 increases antioxidant activity and stability of the tear film and produces anti-inflammatory effect exhibited as downregulation of TNF-*α* and IL-6 and pronounced upregulation of IL-10 in tears. Our data suggest novel features of SkQ1 and point to its feasibility in patients with DES and individuals at risk for the disease including those subjected to general anesthesia.

## 1. Introduction

Dry eye syndrome (DES) is a multifactorial ocular pathology characterized by corneal epithelium lesions, inflammation of ocular surface, and symptoms of discomfort including irritation, itching, and burning eyes [1, 2]. According to the epidemiological studies, DES affects more than 300 million people worldwide and represents the major reason for seeking eye care in developed countries [3]. Ageing, prolonged eye strain, environmental factors, medication intake, and refractive surgery are the major contributors to DES development [4]. General anesthesia is another risk factor for DES, which is becoming more prominent with wider use of surgical interventions in modern medicine [5, 6].

DES is commonly associated with reduced tear production and/or alterations in the tear composition, resulting in the loss of protective and nourishing qualities of tears [1]. The integrity of the outermost layers of the ocular surface is highly dependent on hydration and lubrication, provided by the tear film, as well as on the tear cytokines and growth factors, which promote wound healing and containment of inflammatory responses in the corneal epithelium and stroma. Consistently, current treatment of DES involves usage of lubricating eye drops and ointments or, in more severe cases, anti-inflammatory medication [7]. Unfortunately, therapeutic strategies relying on the moisturization and lubrication of eye surface only provide temporary relief from DES symptoms and have no effect on the pathogenic processes underlying the disease. Treatment with anti-inflammatory drugs, such as steroids, cyclosporine A, and tetracycline, significantly improves clinical state of DES patients. However, prolonged use of corticosteroid eye drops may cause complications, namely, elevated intraocular pressure and cataract, which place restrictions on the duration of such treatment. Cyclosporine instillations cause burning eye sensation, which is a major factor limiting its employment in DES. Antibiotics, such as tetracycline and azithromycin, are applied successfully for the treatment of the disease, but it is strongly recommended to avoid using them at high doses because they are known to cause a number of side effects [8]. Lately, therapeutic application of proteins and peptides has been suggested as a prospective approach to the treatment of DES-associated corneal defects. Yet, such medications are generally based on cytokines, growth factors, hormones, and other naturally occurring tear components and, as such, could produce multifaceted and often contradictory effects on the corneal homeostasis. In addition, the majority of protein-based medications are not yet approved for clinical use [9]. All things considered, the demand for novel approaches to treating DES remains a highly relevant problem in current ophthalmology.

Growing evidence indicates that oxidative stress plays an important role in the pathogenesis of DES [10]. Normally, the tear film provides effective antioxidant protection for the ocular surface. It is enriched in both low molecular weight antioxidants (glutathione, ascorbic acid, and others) and enzymes involved in the replenishment of glutathione pool and first-hand scavenging of reactive oxygen species (ROS) (glutathione reductase, glutathione peroxidase, superoxide dismutase, etc.) [11, 12]. In DES, acute elevation in ROS levels affects corneal epithelial cells directly, by causing irreversible oxidative modifications of nuclear acids, lipids, and proteins, and indirectly, via the increased expression of proinflammatory cytokines. Thus, oxidative stress is known to induce and prolong local inflammatory responses leading to corneal injury [13]. With this in mind, antioxidant preparations to compensate for the loss of intrinsic antioxidant activity might be regarded as a feasible approach to the treatment of DES.

To date, a promising outlook on applying antioxidant therapy for the treatment of DES was demonstrated in experimental and clinical research. Thus, it has been shown that certain antioxidants can suppress inflammation of corneal epithelium and improve lacrimation [14–19]. The positive effect of this therapy could potentially be explained by its ability to balance redox status of tears and corneal epithelium. However, the most potent antioxidants are expected to be those targeting ROS directly in their intracellular sources such as mitochondria [20]. Indeed, intramitochondrial oxidative stress is associated with processes, governing cell survival, such as mitochondrial plasticity, apoptosis, and autophagy [21, 22]. Since the mitochondria are impenetrable to conventional antioxidants, the latter have low effectiveness against ROS formation in these organelles. Thus, a necessity for mitochondria-targeted antioxidants emerged. In the last 15 years, several antioxidants of this class were synthesized, including SkQ1, MitoQ, and SS31 [23–25]. These molecules are able to bypass plasma membrane and outer mitochondrial membrane and accumulate in the mitochondria, thereby displaying exceptional antioxidant activity even in nanomolar concentrations [20, 26]. As a result, they massively outperform all the conventional antioxidants in terms of specificity and activity levels. The employment of this new class of antioxidants represents an attractive pharmacological approach to the therapy of pathologies associated with oxidative stress and peroxidation of proteins and lipids of the inner membrane of mitochondria [20, 26].

Recent studies have demonstrated that administration of mitochondria-targeted antioxidant SkQ1 improves the tear film stability and regeneration of corneal epithelium in polyetiological DES [27, 28]. Furthermore, SkQ1 was found to reduce age-related alterations in lacrimal gland in animals [29]. However, detailed effects of SkQ1 on corneal state and morphology as well as the biochemical mechanisms underlying therapeutic effect of the antioxidant in DES remain scarce. These questions could be addressed using animal models of the disease such as recently characterized rabbit model of anesthesia-induced DES. Indeed, the decline in tear production and tear film stability under general anesthesia leads to erosive processes involving all layers of corneal epithelium, which are highly reminiscent of DES-associated corneal lesions [5]. Furthermore, under these conditions, significant alterations in biochemical and antioxidant properties of tears are observed, similar to those in DES patients [6, 10]. All these features make the rabbit model of anesthesia-induced DES highly convenient for investigating therapeutic strategies for the disease, including antioxidant treatment using SkQ1.

In this work, we employed the experimental model of anesthesia-induced DES to investigate the effect of mitochondria-targeted antioxidant SkQ1 on clinical status, morphology, and biochemical properties of the cornea as well as on the secretion, stability, and protective qualities of the precorneal tear film. It was demonstrated for the first time that the application of SkQ1 results in a prominent improvement of clinical state of the ocular surface by inhibiting apoptotic and necrotic changes in the corneal epithelium. Unexpectedly, this effect was produced not only via suppressing oxidative stress in the cornea but also by stimulating the intrinsic antioxidant defense of this tissue, accelerating the recovery of redox status and integrity of the tear film and dampening the local proinflammatory response. Our data suggest novel mechanisms of SkQ1 action and point to its feasibility in patients with DES and individuals at high risk for the disease including those subjected to general anesthesia.

## 2. Material and Methods

### 2.1. Materials

Anesthetic preparation containing 50 mg/ml tiletamine and 50 mg/ml zolazepam was purchased from Virbac (France). Xylazine hydrochloride was bought from Bioveta (Czech Republic). Fluorescein sodium solution was from Novartis (Switzerland). Ultragrade Tris was purchased from Amresco (USA). Molecular biology grade phosphate buffer saline (PBS) was bought from Gibco. Hemoglobin, luminol, hydrogen peroxide solution, and Trolox (6-hydroxy-2,5,7,8-tetramethylchroman-2-carboxylic acid) were from Sigma-Aldrich (USA). Other chemicals used in this study were from Sigma-Aldrich, Amresco, or Serva (Germany) and were at least of reagent grade. All buffers and other solutions were prepared using ultrapure water. The Schirmer test tear strips were from Contacare Ophthalmics & Diagnostics (India). SkQ1 (10-(6′-plastoquinonyl)decyltriphenylphosphonium) was synthesized and provided by the Institute of Mitoengineering of Moscow State University (Moscow, Russia).

### 2.2. Experimental Animals and Ethics Statement

The study involved a total of 182 healthy pigmented rabbits (6 months old, weight of 2.3 to 3 kg) purchased from a certified farm (Krolinfo, Russian Federation). The rabbits were housed at a 12 h light-dark cycle at a temperature of 22–25°C and humidity of 55–60% with free access to food and water. The animals' treatment was performed according to the 8th edition “Guide for the Care and Use of Laboratory Animals” of the National Research Council and “Statement for the Use of Animals in Ophthalmic and Visual Research” of The Association for Research in Vision and Ophthalmology (ARVO). The protocol was approved by the Belozersky Institute of Physico-chemical Biology Animal Care and Use Committee (Protocol number 1/2016).

The experiments were performed using a single-blind method. The rabbits were divided into 29 (22 experimental and 7 control) groups of 6–8 animals (see supplementary Table S1 available online at https://doi.org/10.1155/2017/9281519) and treated as described in Results. To assess safety of SkQ1 administration in a form of eye drops, the animals were medicated by conjunctival instillations of 50 *μ*l of either vehicle solution (7 mM NaH_2_PO_4_, 2.6 mM Na_2_HPO_4_·12H_2_O, containing 150 mM NaCl, 0.0001% benzalkonium chloride) or the same solution containing 0.25, 2.5, 7.5, or 25 *μ*M SkQ1 (SkQ1 eye drops) in each eye 3 times a day for 7–30 days. To induce general anesthesia, the animals were placed in prone position in a restraining device and subjected to intramuscular injection of anesthetic preparation containing 50 mg/ml tiletamine and 50 mg/ml zolazepam. The injections were repeated pro re nata to achieve continuous narcotic sleep of the required duration (see supplementary Table S1). Antioxidant premedication was performed as follows. On the day before anesthesia, the animals received conjunctival instillations of either vehicle solution or the same solution containing 0.25 *μ*M, 2.5 *μ*M, or 7.5 *μ*M SkQ1, 1 drop 3 times a day. On the next day, an additional instillation of the vehicle/total solution was carried out, and the animals were exposed to general anesthesia. For the antioxidant therapy, the animals were firstly exposed to general anesthesia and then they received conjunctival instillations of the vehicle solution or the same solution containing 0.25 *μ*M, 2.5 *μ*M, or 7.5 *μ*M SkQ1, 1 drop 3 times a day, for 30 days, starting from the moment of recovery from the narcosis. In the course of all experiments, the animals were kept under normal conditions described above. After the characterization of corneal state and/or tear collection, the animals were rehabilitated for 3 days and returned to the farm. For biochemical and histological studies of the cornea, the rabbits were humanely euthanized by introduction into general anesthesia and subsequent intracardiac injection of the 1 ml of 20 mg/ml xylazine hydrochloride. Enucleating of the eyeballs and corneal excision were performed postmortem.

### 2.3. Clinical Examination of the Cornea

The development of corneal injury was monitored by fluorescein staining of the ocular surface [30]. Briefly, 2 *μ*l of 1% sodium fluorescein was instilled under the lower eyelid of the animal and distributed over the cornea by 2-3 movements imitating eye blinking. The corneal status was then examined under the slit-lamp microscope with or without cobalt blue filter. The registered corneal injuries were assigned with clinical scores (CS) of 0–4 depending on the size of the affected corneal surface (fluorescein rating scale): no fluorescein staining (CS = 0), staining of 0–12.5% of the corneal surface (CS = 1), staining of 12.5–25% of the corneal surface (CS = 2), staining of 25–50% of the corneal surface (CS = 3), and staining of >50% of the corneal surface (CS = 4). Mean clinical scores (MCS) were derived by adding the clinical scores for all eyes of the animals in a group and dividing by the number of eyes ± standard deviation (SD). Incidence of DES was determined as percent of eyes with diagnosed corneal injury regardless of its score.

### 2.4. Histological Analysis

The animals were sacrificed after fluorescein staining of the cornea given that the fluorescein exposure does not impact tissue morphology [31, 32]. The eyeballs were enucleated immediately postmortem and fixed in 10% neutral buffered formalin in phosphate buffer (pH 7.4) for 24 hours at room temperature or in Carnoy's solution (60% ethanol, 30% chloroform, and 10% glacial acetic acid) for 3 hours at room temperature. The corneas and irises were trimmed out of the fixed eyeballs along the corneal limbus, dehydrated by incubation in absolute isopropanol for 5 h and embedded in Histomix paraffin medium. Ten four-micron thick nasotemporal cross-sections of the cornea with iris were prepared and mounted on glass slides. The sections were then deparaffinized (xylene, 5 minutes, two times), placed to absolute isopropanol (5 minutes, two times), hydrated, and stained with Carazzi's hematoxylin and 0.5% eosin Y in water or with periodic acid and Schiff (PAS) reagent [33]. Stained sections were dehydrated by 96% ethanol and xylene, cleared in BioClear tissue clearing agent, mounted into BioMount synthetic medium, and examined using an Axio Scope.A1 microscope (Carl Zeiss, Germany). Lesions in the cornea were assigned with severity scores of 0–5, representing unremarkable, slight, mild, moderate, marked, and severe, respectively. Mean severity scores (MSS) were derived by adding the severity scores for all animals (eyes) in each treated group and by dividing by the number of animals (eyes) in the group [34]. Incidence of the lesions was determined as a percent of eyes with diagnosed lesion regardless of its score. Microphotographs were obtained by an AxioCam MRc 5 megapixel color camera (Carl Zeiss, Germany) and processed using AxioVision v.3.0 (Carl Zeiss, Germany) and Photoshop CS3 software (Adobe Systems, USA). For the safety study, the lens and optic nerve were histologically processed and microscopically examined as described above. The retina was analyzed as described previously [35].

### 2.5. Schirmer's Test

Tear secretion was measured by means of standardized the Schirmer test [36]. Briefly, gauged Schirmer's test paper strips were placed under the lower eyelid and removed after 5 minutes of soaking, and the length of the moistened paper (in mm) was recorded. The procedure was repeated at least three times, and the average values were considered.

### 2.6. BUT Test

Stability of the tear film was examined using standard tear film breakup time (BUT) test [37]. The instillations of fluorescein into the rabbit eye were performed exactly as for clinical examination of the cornea (see above). After each instillation, the time of appearance of the first dry spot in the central cornea seen under a slit-lamp microscope was measured.

### 2.7. Corneal and Tear Samples

To obtain corneal homogenates, the full-size rabbit corneas were excised, placed into 400 *μ*l of PBS, and frozen at −70°C. After thawing, the homogenate was sonicated for 10 min on ice. Corneal extracts for biochemical evaluations were obtained by centrifugation of the homogenates (15000*g*, 10 min) at +4°C. The tear samples were collected using gauged Schirmer's test strips. To this end, the length of the moistened strip was allowed to reach exactly 20 mm and its 15 mm fragments were cut off and extracted with 150 *μ*l of PBS.

### 2.8. Malondialdehyde Assay

MDA concentration was measured in corneal extracts using commercially available kits (Sigma-Aldrich, USA) following the manufacturer's instructions. Intensity of colorimetric reaction was determined using MR-96A Microplate Reader (Mindray, China). The data were analyzed using SigmaPlot 11 (SYSTAT Software).

### 2.9. Total Protein Concentration

Protein concentration in corneal extracts and tear samples was measured by the bicinchoninic acid (BCA) method using a BCA protein assay kit (Thermo Fisher Scientific, USA) in accordance with the manufacturer's instructions.

### 2.10. Total Antioxidant Activity

The corneal and tear samples were analyzed using hemoglobin-H_2_O_2_-luminol model system [38]. Standard solutions, containing 1–8 *μ*M Trolox in PBS, were used as a reference. Thirty microliters of standard solution or tear samples diluted 1 : 4 by PBS was added to 0.44 ml of reaction mixture, containing 0.01 mM luminol and 0.5 mM hemoglobin in PBS. Luminol oxidation reaction was stimulated by the addition of hydrogen peroxide to reach 6 *μ*M. Chemiluminescence of the sample was registered each 1 second for 10 minutes using Glomax-Multi Detection System luminometer (Promega, USA). The data were analyzed using SigmaPlot 11 (SYSTAT Software, USA), and total antioxidant activity (AOA) was expressed in Trolox equivalent.

### 2.11. Antioxidant Enzyme Activity

The activity of tear enzymes involved in antioxidant protection (superoxide dismutase (SOD), glutathione peroxidase (GPx), glutathione reductase (GR), and glutathione-SH transferase (GST)) was evaluated in the corneal and tear samples, using commercially available kits (Sigma-Aldrich, USA) in accordance with the manufacturer's instructions. Intensity of colorimetric reactions was determined using MR-96A Microplate Reader (Mindray, China). The acquired data were analyzed using SigmaPlot 11 (SYSTAT Software). The activity of the enzymes in corneal extracts was normalized to 1 mg of the total protein.

### 2.12. Inflammatory Cytokine Content

The content of tumor necrosis factor alpha (TNF-*α*) and interleukins 4 (IL-4), 6 (IL-6), and 10 (IL-10) was examined in the tear samples using Rabbit ELISA kits (Cusabio Biotech, China) according to the protocols provided by the manufacturer. The colorimetric reaction was digitalized using MR-96A Microplate Reader (Mindray).

### 2.13. Statistics

The data were analyzed by the mean standard deviation method, using SigmaPlot 11 Software (SYSTAT Software). The bars in the figures represent standard deviation (SD). Mean scores, SD, and statistical significance were calculated using SigmaPlot 11 Software or BioStat 2009 version 5.8.3.0 software (AnalystSoft, USA). Statistical significance was assessed using paired *t*-test (for safety evaluations) or unpaired two-tailed *t*-test. The probability of 0.05 was considered significant.

## 3. Results

### 3.1. Safety of SkQ1 Eye Drop Administration

Since SkQ1 is a nonnatural antioxidant, we firstly studied whether repetitive conjunctival instillations of SkQ1 eye drops can induce ocular toxicity in rabbits. To this end, the animals were medicated with the solution containing 0.25, 2.5, 7.5, or 25 *μ*M SkQ1 during one week and their eyes/eye tissues were examined visually (lids, lacrimal apparatus, and conjunctiva), ophthalmoscopically (cornea, anterior chamber, lens, iris, and vitreous humor), and histologically (cornea, lens, iris, retina, and optic nerve). It was found that administration of SkQ1 eye drops was generally well tolerated at a dosage of 0.25, 2.5, and 7.5 *μ*M SkQ1. Instillations of 25 *μ*M SkQ1 induced conjunctival redness in one animal. Thus, the dose of 0.25, 2.5, and 7.5 *μ*M SkQ1 was considered safe for the further applications. Given that the main hallmark of DES is the altered tear secretion, we assessed this parameter in animals before and after the instillation of 0.25, 2.5, or 7.5 *μ*M SkQ1 3 times a day for 1 month. The divergence of the results of the Schirmer test did not exceed 15% (*p* > 0.05) in all cases, indicating almost no effect of the drug on the tear secretion (Table 1).

### 3.2. Experimental Model of General Anesthesia-Induced DES for Trialing of SkQ1 Treatment

**Figure 1:**
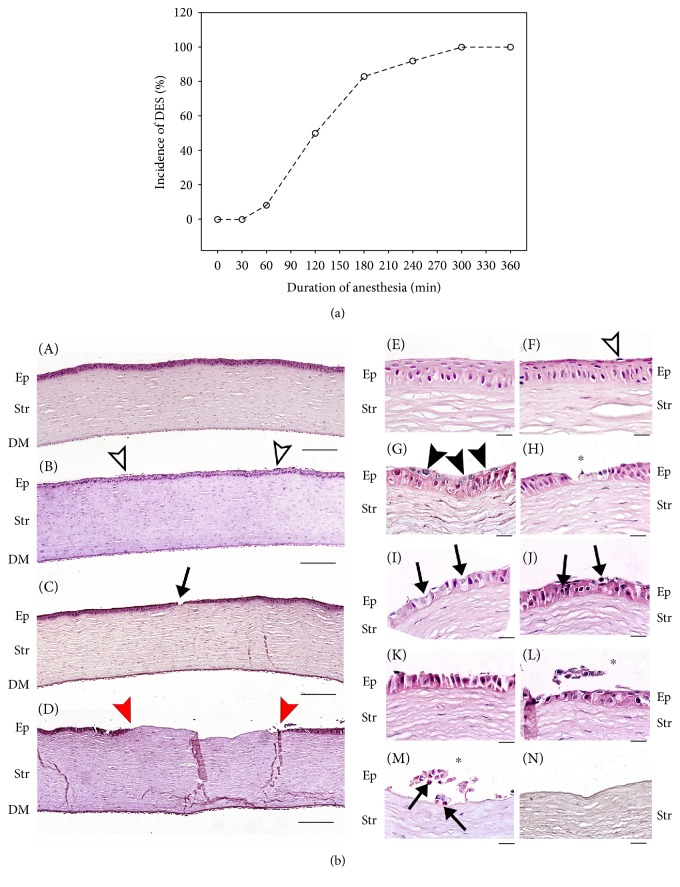
Clinical state and morphology of the cornea during general anesthesia. (a) Incidence of DES in the rabbits exposed to 1–6 h of general anesthesia calculated as a percent of eyes with fluorescein-stained corneal injury regardless of its score. (b) Representative microscopic images of hematoxylin and eosin staining of rabbit corneas from different experimental groups. Control animals: normal cornea (А, Е). Animals after 1 h of general anesthesia: desquamation of superficial epithelial cells is indicated by white arrowhead (B, F). Animals after 3 h of general anesthesia: degeneration of epithelial cells is shown by black arrowheads (G), destruction of epithelial layer is indicated by an arrow (C), and the same locus is shown at higher magnification by an asterisk (H). Animals after 6 h of general anesthesia: prenecrotic (I) and apoptotic (J) changes are indicated by black arrows, desquamated cornea epithelial cells onto partially or fully thickness-denuded area are shown by asterisks (L-M), apoptotic degeneration of some cells is shown by arrows (M), and locus of total denudation is shown by arrowheads at lower (D) and higher (N) magnification (margins of cornea erosion are shown by red arrowheads). Ep: epithelium; Str: stroma; DM: Descemet's membrane and corneal endothelium. Magnification: ×100 (A–D) and ×1000 (E–N); scale bars: 200 *μ*m (A–D) and 20 *μ*m (E–N).

A rabbit model of anesthesia-induced DES developed in our previous studies [6, 39] was selected for further trialing of the antioxidant therapy using SkQ1 eye drops. In order to characterize the basic parameters of the model, we determined the incidence of DES symptoms in rabbits depending on the duration of general anesthesia. To this end, the animals were exposed to general anesthesia for 0.5-6 h and their corneas were subjected to clinical and morphological examination immediately after the narcosis. The results of fluorescein test indicated anesthesia time-dependent accumulation of corneal epithelium injury in accordance with our previous findings [39]. Statistical analysis revealed that the incidence of the corneal damage rose with the duration of the narcotic sleep reaching 100% of animals after 5-6 h of the anesthesia (Figure 1(a)). Similar observations were made upon histopathological examination of corneal samples obtained from animals exposed to 1 h, 3 h, and 6 h of anesthesia (Figure 1(b), Table 2), while in control rabbits, the cornea displayed normal structure (Figure 1(b), A, E), and in 1 h anesthetized animals, it exhibited a loss of superficial epithelial layer as well as coagulation, desquamation, and shedding of the squamous cells (Figure 1(b), B, F). Exposure to 3 h anesthesia resulted in focally more extensive corneal injuries such as degenerative and necrobiotic alterations in outer wing cells denuded after superficial cell loss (Figure 1(b), C, G). These multifocal epithelial defects included cytoplasmic changes (swelling and lightening) and nuclear changes (enlargement and lightening) (Figure 1(b), G, arrows), that corresponded to typical hydropic degeneration [40]. In some cases, the injured wing cells were separated from the inner layers with only one layer of degenerating basal cells remaining at the damaged locus (Figure 1(b), H, asterisk). After 6 h of anesthesia, the corneal injuries became much more severe and diffused (Figure 1(b), D). Hydropic cell degeneration was more pronounced and detected in both wing and basal cells (Figure 1(b), I). Furthermore, progressive cell loss was accompanied by signs of apoptosis that include cell shrinkage, chromatin condensation, nuclear changes (pyknosis or karyorrhexis), dense staining, and the formation of apoptotic bodies (Figure 1(b), I-J, arrows). In addition, separated cells at different morphological stages of necrotic (Figure 1(b), L, asterisk) or apoptotic (Figure 1(b), M, arrows) cell death were found. Full-thickness epithelial damage resulted in the disruption of basal cell adhesion to the basal lamina, total loss of epithelial layer, and denudation of underlying stroma (Figure 1(b), N). The most pronounced areas of denudation in 6 h treated animals were more than 1 mm in diameter (Figure 1(b), D). Overall, by the end of 3–6 h general anesthesia, the full-scaled pathomorphological picture of DES was developed in the majority of the animals. Considering that exposure to anesthesia of similar duration reduces tear secretion significantly [6], the condition developed after 6 h narcosis was recognized as a model of severe DES, which is feasible for the proper trialing of the proposed antioxidant therapy.

### 3.3. Efficacy, Dosage, and Administration Scheme for SkQ1 in Treatment of General Anesthesia-Induced DES

**Figure 2:**
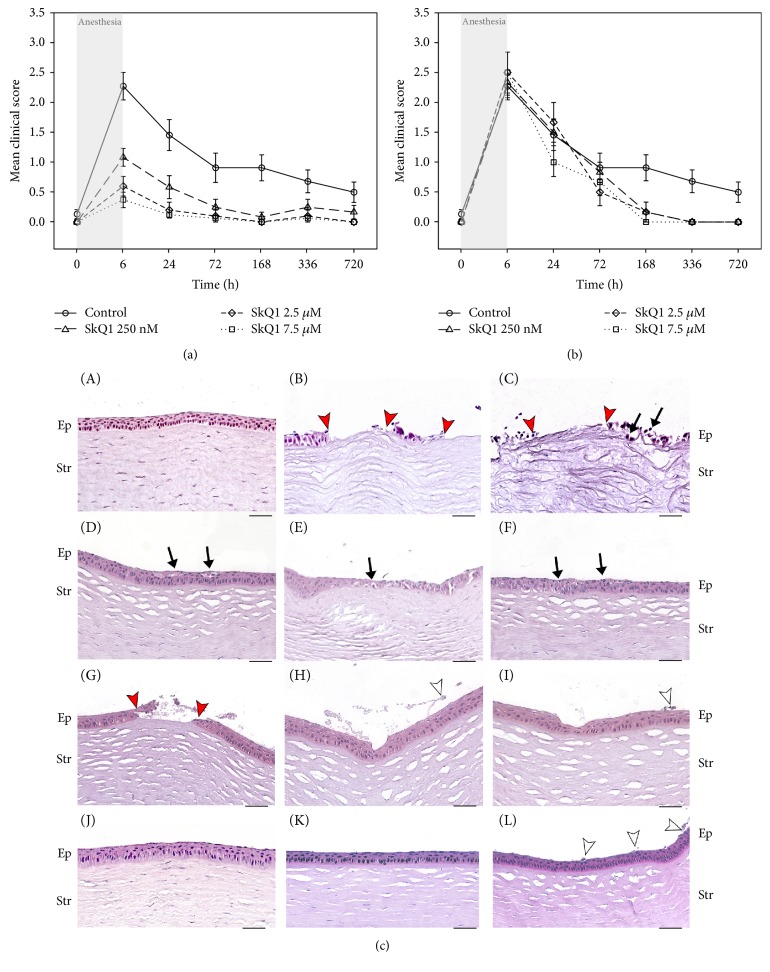
Clinical state and morphology of the cornea in general anesthesia-induced DES upon premedication or treatment with SkQ1. Corneal status in the rabbits with anesthesia-induced DES premedicated (a) or treated (b) with SkQ1 eye drops. *p* < 0.05 for all values measured in all premedication groups and in 7.5 *μ*M SkQ1 treatment group compared with the values obtained for the control group. (c) Representative microscopic images of hematoxylin and eosin staining of rabbit corneas from different experimental groups. Control animals: normal cornea (А, J). Cornea immediately after 6 h general anesthesia: without premedication (B, C) and after premedication with 0.25 (D, E), 2.5 (F, G), or 7.5 *μ*M (H, I) SkQ1. Cornea after 6 h of general anesthesia on the fourteenth day of treatment with 7.5 *μ*M SkQ1 (K, L). Margins of cornea erosion are shown by red arrowheads; desquamation of superficial epithelial cells is indicated by white arrowheads; prenecrotic and apoptotic changes are indicated by black arrows. For abbreviations see Figure 1. Magnification: ×400; scale bar 50 *μ*m.

To assess the efficacy of SkQ1 eye drops for the treatment of anesthesia-induced DES, the experimental animals were treated according to the following alternative schemes. The animals were either premedicated using instillations of SkQ1 at a concentration of 0.25, 2.5, or 7.5 *μ*M prior to induction of DES or medicated with the same dosage of the antioxidant starting immediately after narcosis and continuing during one month after the induction of the disease. In both cases, the clinical state of the cornea was examined by fluorescein test immediately after the narcosis and on the 1st, 3rd, 7th, 14th, and 30th days of the subsequent time period. In addition, some of the animals were withdrawn right after the anesthesia (for premedicated groups) or on the 14th day of the postanesthetic period (for treated groups) for histological and biochemical studies of the cornea. As it can be seen from Figure 2(a), SkQ1 premedication had a pronounced protective effect on the cornea that was enhanced with an increase of the drug dosage. Thus, 7.5 *μ*M SkQ1 prevented pathological changes in the cornea after recovering from the narcosis and completely neutralized clinical signs of DES as early as the first day of the postanesthetic period. By contrast, the treatment by the antioxidant after the induction of DES was less effective and less dose-dependent, although a complete recovery of the corneal injuries was also accelerated becoming reduced to one week (Figure 2(b)). These observations are generally in accord with the results of histological examination of the corneas (Figure 2(c)). Thus, in contrast to the untreated animals (Figure 2(b), B-C), the animals premedicated with eye drops containing different concentrations of SkQ1 exhibited no foci of total destruction of the epithelium by the end of the anesthesia (Figure 2(c), D–I). Moreover, no prenecrotic and necrotic changes and no cells with enlarged and enlightened cytoplasm were found in such animals. The minor changes in this case included the presence of single apoptotic cells (Figure 2(c), C–F) and consequent reduction of corneal epithelium thickness in some areas (Figure 2(c)). The least decrease of the epithelium thickness was found following administration of 7.5 *μ*M SkQ1 (Figure 2(c), H-I). By contrast, in the animals medicated with the same dosage of SkQ1 after anesthesia, the morphology of the corneal epithelium returned to the normal state only at the fourteenth day of the postanesthetic period (Figure 2(d), K-L). It should be added that by that time the signs of active reepithelialization were absent, assuming that this process was already completed.

We concluded that SkQ1 possesses a positive effect on corneal state and that this effect is more pronounced when the drug is administrated prior to anesthesia-induced DES. Thus, SkQ1 more likely exhibits protective action on healthy corneal epithelium, rather than participate in the corneal wound healing. Since the most prominent protective effect was observed in the case of premedication using 7.5 *μ*M SkQ1, this dosage and administration scheme was considered optimal for reproducing in subsequent experiments.

### 3.4. Oxidative and Antioxidant State of the Cornea in General Anesthesia-Induced DES with or without SkQ1 Premedication

**Figure 3:**
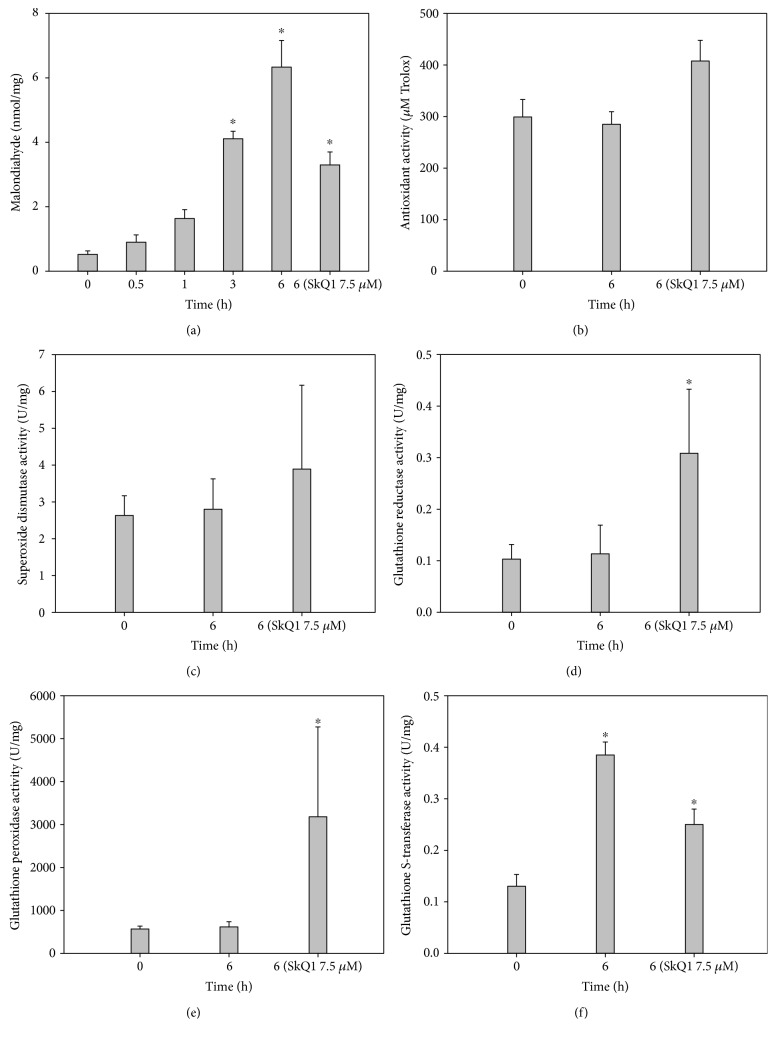
Oxidative stress and antioxidant activity in the cornea in general anesthesia-induced DES upon premedication with SkQ1. (a) Concentration of MDA in rabbit corneal homogenates after 1–6 h of general anesthesia with or without premedication using 7.5 *μ*M SkQ1. (b)–(f) Total antioxidant activity (b) and activity of SOD (c), GR (d), GPx (e), and GST (f) in corneal extracts after 6 h of general anesthesia with or without premedication using 7.5 *μ*M SkQ1. ^∗^*p* < 0.05 compared with the values measured in control group before general anesthesia.

The simple explanation of the revealed positive effect of SkQ1 premedication in anesthesia-induced DES was its direct antioxidant action on corneal epithelial cells. With that in mind, we compared oxidative stress and intrinsic antioxidant activity in the corneas of the control and SkQ1-premedicated animals. Without premedication, the concentration of malondialdehyde (MDA) in corneal homogenates increased noticeably with the duration of anesthesia reaching more than 10-fold excess by the 6th hour of the narcosis (Figure 3). By contrast, the antioxidant premedication using 7.5 *μ*M SkQ1 attenuated the increase in the MDA concentration after 6 h anesthesia by 50%. This effect can be partially associated with an increase in the antioxidant protection in the cornea due to its amelioration by the drug action. Indeed, without antioxidant treatment, the animals with anesthesia-induced DES exhibited no noticeable changes in AOA, SOD activity, and glutathione-metabolizing enzymes (GR and GPx) in corneal extracts (Figures 3(b), 3(c), 3(d), and 3(e)). An exception was the growth of GST activity that may be related to the detoxification of the injected anesthetic (Figure 3(f)). By contrast, the animals premedicated with 7.5 *μ*M SkQ1 demonstrated a significant increase in activities of GR and GPx, which were higher than the respective values registered both in the animals with untreated DES (Figures 3(d) and 3(e)). In the case of GPx, the effect was the most pronounced reaching a 5–10-fold excess of the baseline control value (Figure 3(e)). Finally, the increase in GST activity was attenuated in the cornea of SkQ1-treated animals (Figure 3(f)). Thus, the anesthesia-induced DES was associated with oxidative stress of the corneal cells without a compensatory increase in their own antioxidant protection. Under these conditions, SkQ1 possessed prominent protective activity both by inhibiting oxidative stress in the cornea and upregulating its intrinsic antioxidant defense enzymes, foremost GPx and GR.

### 3.5. Tear Secretion and Stability in General Anesthesia-Induced DES with or without SkQ1 Premedication

**Figure 4:**
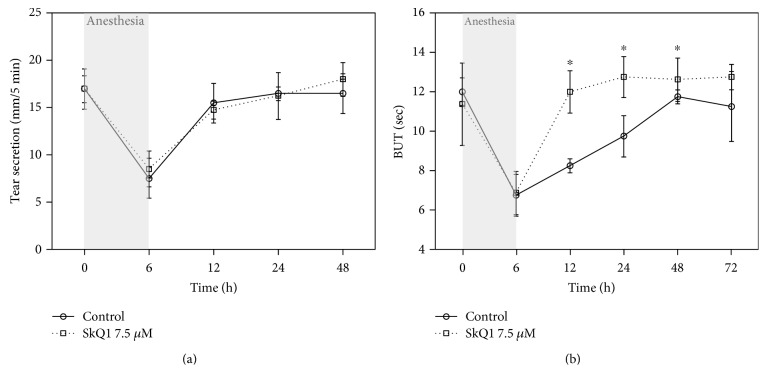
Secretion and stability of the tear film in general anesthesia-induced DES upon premedication with SkQ1. Results of standardized Schirmer's (a) and BUT (b) tests performed in the rabbits exposed to 6 h of general anesthesia with or without premedication using 7.5 *μ*M SkQ1. ^∗^*p* < 0.05 compared with the values measured in control group.

In addition to the direct effect on the corneal cells, SkQ1 may positively affect tear-producing ocular tissues and improve tear quantity and/or quality thereby producing the revealed protective effect on the cornea. To test this suggestion, we further examined the effect of premedication using 7.5 *μ*M SkQ1 on the tear secretion and stability in rabbits with anesthesia-induced DES by means of standardized Schirmer's and BUT tests, respectively. Exposure of the animals to general anesthesia for 6 h resulted in 2.3-fold reduction of their tear secretion, which returned to the normal values by 12 h of the postanesthetic period regardless of SkQ1 administration (Figure 4(a)). Meanwhile, the decrease in tear stability in the control animals recovered much slower (48 h), whereas the antioxidant premedication significantly accelerated the recovery (Figure 4(b)). Thus, SkQ1 had no effect on the tear secretion, but may alter tear composition thereby increasing stability and protective features of the tear film.

### 3.6. Antioxidant Activity of the Tear Fluid in General Anesthesia-Induced DES with or without SkQ1 Premedication

**Figure 5:**
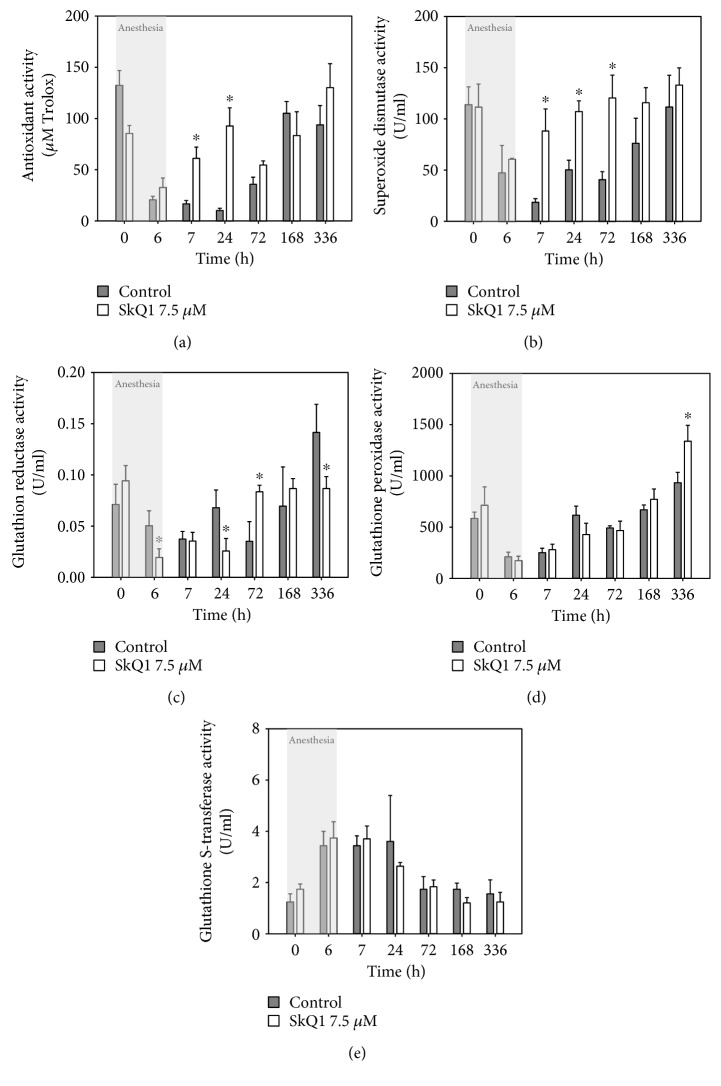
Antioxidant activity of the tear fluid in general anesthesia-induced DES upon premedication with SkQ1. Total antioxidant activity (a) and activity of SOD (b), GR (c), GPx (d), and GST (e) rabbit tear samples after 6 h of general anesthesia with or without premedication using 7.5 *μ*M SkQ1. ^∗^*p* < 0.05 compared with the values measured in control group.

As the next step, we monitored the effect of SkQ1 on antioxidant properties of the tear in the animals with anesthesia-induced DES. Tear samples were collected from control and 7.5 *μ*M SkQ1-premedicated rabbits prior to 6-hour general anesthesia, immediately after the recovery from narcosis or following 1 hour, 24 hours, 3 days, 1 week, or 2 weeks of the postanesthetic period. It was found that the development of the corneal injury in the control animals was associated with a pronounced decrease in AOA and the activity of antioxidant enzymes in their tear fluid. In such self-limited DES, these parameters restored only in 1-2 weeks after the narcosis (Figure 5). The premedication using SkQ1-containing eye drops had no effect on the total protein concentration in tears (data not shown), but it accelerated significantly the normalization of AOA and SOD activities (Figures 5(a) and 5(b)). Indeed, in the SkQ1-treated animals, these parameters returned to their baseline values as early as the first day of the postanesthetic period. Thus, the revealed therapeutic action of SkQ1 in anesthesia-induced DES may include protection of the tear-producing tissues and associated improvement of the antioxidant activity of the tear film.

### 3.7. Inflammatory Cytokines of Tear Fluid in General Anesthesia-Induced DES with or without SkQ1 Premedication

**Figure 6:**
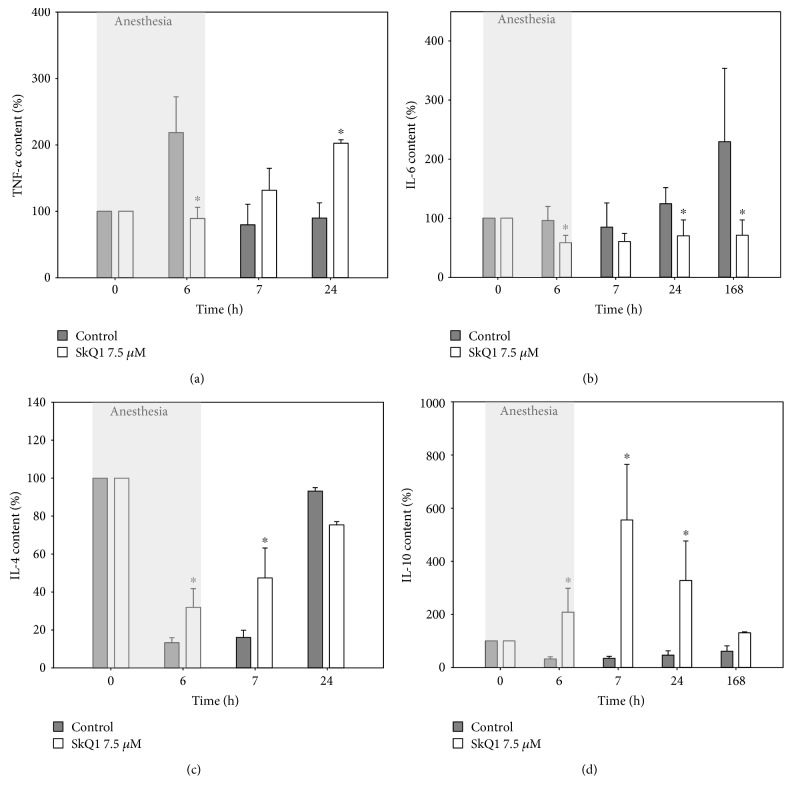
Inflammatory cytokines in the tear fluid in general anesthesia-induced DES upon premedication with SkQ1. Concentration of TNF-*α* (a), IL-6 (b), IL-4 (c), and IL-10 (d) rabbit tear samples after 6 h of general anesthesia with or without premedication using 7.5 *μ*M SkQ1. ^∗^*p* < 0.05 compared with the values measured in control group.

It is widely regarded that DES pathogenesis involves inflammatory component [9, 39]. The oxidative stress of the cornea detected in our DES model can induce inflammatory responses of the ocular surface tissues [41] that may be sensitive to SkQ1 action. To test this assumption, we examined profiles of the common tear cytokines in the 6 h anesthetized rabbits with or without premedication using 7.5 *μ*M SkQ1 (Figure 6). Approximately a 2-fold increase in proinflammatory cytokines was observed in the control animals with DES, namely, the fast growth of TNF-*α* concentration (within 6 hours) followed by the delayed growth in IL-6 (Figures 6(a) and 6(b)). These effects were associated with a prominent reduction in anti-inflammatory cytokines IL-4 and IL-10 (Figures 6(c) and 6(d)). Without treatment, TNF-*α* and IL-4 restored to their normal levels within 1 h and 1 day, respectively (Figures 6(a) and 6(b)). In contrast, premedication using 7.5 *μ*M SkQ1 considerably suppressed the release of both proinflammatory cytokines and accelerated the recovery of IL-4. Interestingly, it produced a pronounced stimulatory effect on the secretion of IL-10, the concentration of which reached ~550% of the normal value within an hour after anesthesia (Figure 6(d)). Overall, the protective effect of SkQ1 may involve the inhibition of inflammatory responses of the ocular surface via suppression of the preceding oxidative stress and/or by specific overstimulation of secretion of anti-inflammatory cytokine IL-10.

## 4. Discussion

The goal of this study was to determine the safety, efficacy, and optimal dosage regimen of administration of mitochondria-targeted antioxidant SkQ1 for prevention and treatment of DES and to suggest cellular and biochemical mechanisms underlying therapeutic action of SkQ1. To this end, it was necessary to monitor alterations in morphological and biochemical properties of the cornea, as well as biochemical and biomechanical parameters of tears, under the course of the disease. However, performing these experiments in humans was hindered by limited availability of the patients for ophthalmological examination and by impossibility of obtaining the required tissue samples. Considering these limitations, we employed the rabbit model of the disease since these species share many features of ocular anatomy with humans including eyeball size, its internal structure, and optical system as well as biomechanical and biochemical features [42]. DES was simulated in rabbits by their exposure to general anesthesia [6, 39, 41]. Indeed, anesthetic injections are known to inhibit lacrimal gland innervations and suppress tear production [43]. These complications are often observed in humans during and after surgery, leading to manifestation of iatrogenic corneal lesions or so-called perioperative DES. Yet, the exposure of rabbits to general anesthesia resulted in the development of the majority of symptoms characteristic of common age-related DES, which makes such a model appropriate for testing general approaches to treatment of this disorder [5].

To optimize and characterize the selected model, we monitored time-dependent development of corneal epithelium injury on both clinical and histological levels. Fluorescein-stained point lesions became prominent as early as 1 h postinjection of the anesthetic, whereas after 5-6 h of general anesthesia the corneal injuries developed in all animals. According to the histological study, they were caused generally by cell desquamation or degeneration, cell death (necrotic and apoptotic variants), and separation of the dying cells from the basement membrane. The injuries exhibited as marked attenuation or thinning of epithelium or its total loss (denudation) [34]. However, they did not extend into the stroma thereby representing corneal abrasion (erosion), but not corneal ulcer [44]. The signs of apoptotic and necrotic cell death were detected in middle and deep layers of corneal epithelium most commonly after 6 h of general anesthesia. By this time, the animals additionally exhibited alterations in biochemical properties of tears, in particular, decline of redox status and anti-inflammatory qualities of the tear film and loss of its stability. Thus, after 6 h of general anesthesia, the full-scale picture of DES, similar to the one found in patients [45], can be observed in experimental animals. These results provided the rationale for using this model in the treatment studies.

Like most age-related disorders, DES is associated with development of oxidative stress, which makes antioxidant therapy a favorable approach to its treatment. The feasibility of such strategy was demonstrated previously in both experimental models and clinical studies with a wide range of antioxidants, including alpha-lipoic acid, blueberry components, essential omega-3 fatty acids, L-carnitine, and selenium compounds [14–19]. However, despite their therapeutical efficiency, these chemicals have a common limitation, namely, their inability to suppress ROS formation in mitochondria [22]. In addition to providing cells with energy, the mitochondria take part in the regulation of many signaling cascades, triggered by the oxidative stress, and remain as the major source of ROS [46]. Correspondingly, in many disorders and pathological syndromes, the mitochondria are viewed as attractive targets for therapy [47–49]. Since mitochondria are impermeable to natural (conventional) antioxidants, two novel approaches to their targeting were developed recently [23–25]. Firstly, specific peptide structures were designed to penetrate into mitochondria thereby delivering protein-based antioxidants. Secondly, membrane-permeable cations were created to transport low molecular weight antioxidants to these organelles. The latter compounds demonstrated their efficacy in, for instance, preventing ROS-induced modifications of cardiolipin, the key phospholipid component of mitochondria and a common target of oxidation [50, 51]. In the current study, we used mitochondria-targeted antioxidant SkQ1 (10-(6′-plastoquinonyl)decyltriphenylphosphonium), which was designed previously using the second approach. It is comprised of naturally occurring antioxidant plastoquinone, conjugated with transport molecule triphenylphosphonium, which ensures the penetration of the drug into the cell and its accumulation in the mitochondria [23].

All mitochondria-targeted antioxidants developed so far, including SkQ1, are purely synthetic with no natural analogues. As such, it is important to examine thoroughly their toxicological properties before administering them to the patients. It is known that topical application of pharmaceuticals typically excludes unwanted systemic effects on the organism. In ophthalmology, this can be achieved by using eye drops. Our safety tests reveal that 3 instillations of SkQ1 per day are well tolerated in animals when concentration of the antioxidant did not exceed 7.5 *μ*M, which was recognized as the maximum admissible dose. We further demonstrated protective activity of SkQ1 towards the cornea in anesthesia-induced DES on clinical and morphological levels. The premedication with SkQ1 produced dose-dependent effects in the 0.25–7.5 *μ*M range. Consistently, dose-dependence of SkQ1 action was demonstrated in patients with polyetiological DES [27, 28]. It is noteworthy that SkQ1 had poor dose-dependency when used in treatment after anesthesia. Apparently, premedication with higher dose of the antioxidant results in its faster and more effective accumulation in the cornea, which is less essential in the case of continuous long-term treatment. Overall, regular prophylactic instillations of 7.5 *μ*M SkQ1 can be considered as safe and effective preventive care strategy for DES.

As the next step, we attempted to specify cellular and biochemical mechanisms underlying therapeutic action of SkQ1 in DES. Our histological studies demonstrated that premedication with the antioxidant prevented corneal injury by inhibiting necrotic, prenecrotic, and apoptotic changes in the corneal epithelium. The similar protective activity of SkQ1 was demonstrated for a number of tissues. For instance, it exhibited a noticeable protective effect on heart and kidney infarction caused by ischemia/reperfusion, as well as on ischemic stroke or renal failure [52, 53]. In addition, antiapoptotic activity of SkQ1 towards neurons was demonstrated in the models of Alzheimer's disease and age-related retinopathy [54–57]. The simplest possible explanation of the protective effect of SkQ1 on the cornea is that its accumulation in the mitochondria of corneal epitheliocytes suppresses generation of ROS and propagation of oxidative stress. Indeed, the application of SkQ1 reduced the amount of malondialdehyde in corneal homogenates indicating decrease of oxidative stress in the cornea. Unexpectedly, SkQ1 also stimulated intrinsic antioxidant defense of this tissue. Two major antioxidant systems are distinguished in the cornea and other tissues. Nonenzymatic antioxidants (e.g., vitamins C and E) can provide direct protection from oxidative damage and enhance endogenous antioxidant enzyme activity via synergic scavenging of free radicals [58]. Enzymatic antioxidant system is comprised of superoxide dismutases (cytoplasmic Cu/Zn-SOD (SOD1) and mitochondrial Mn-SOD (SOD2)), catalase, glutathione peroxidases (GPx), and glutathione reductase (GR) [59]. SOD reduces superoxide radicals to hydrogen peroxide, which is decomposed into oxygen and water by catalase or (with oxidation of glutathione) by GPx. Inactivation of these enzymes leads to accumulation of peroxide and its eventual conversion into highly toxic hydroxyl radical. Finally, GR is able to reduce oxidized glutathione, replenishing its necessary pool [60–63]. We have found that premedication with SkQ1 increases GPx and GR levels in the cornea, while having almost no effect on low molecular antioxidant activity (AOA) and SOD activity in this tissue. It should be mentioned that anesthetic conditions by themselves did not cause alterations in GPx and GR activity and the observed trends might be attributed to SkQ1 treatment. The obtained data agree with the effect of another mitochondria-targeted antioxidant MitoQ, which was demonstrated to induce GPx1 expression *in vitro* in leukocytes isolated from the second-type diabetic patients [64]. Yet, our data is the first demonstration of such effect of mitochondria-targeted antioxidant *in vivo*. Overall, application of SkQ1 provides dual action on corneal state in DES by both neutralizing ROS directly and enforcing the key elements of the antioxidant defense.

Normal maintenance of the ocular surface relies on the tear film, which prevents the cornea from desiccative stress and infection and provides it with nourishing and antioxidant compounds [65]. Consistently, in our previous studies, we have shown that development of anesthesia-induced DES in experimental animals is accompanied by a decline in tear film stability as well as by reduction in its antioxidant and anti-inflammatory activity [6]. Such alterations were found in patients with DES and suggested to play the key role in its pathogenesis [10]. Remarkably, administration of SkQ1 in general anesthesia-induced DES not only affected cornea state but also improved tear qualities. In particular, it attenuated a decline in total AOA and SOD. Meanwhile, no substantial change in GPx and GR activities was observed, in contrast to the alterations observed in the cornea. This divergence can be tied to the fact that the antioxidant components accumulate in corneal tissue and tear fluid via distinct mechanisms. In particular, SOD and low molecular antioxidants are secretable compounds therefore entering tear fluid [58]. It is known that DES can be induced by denervation of the lacrimal glands, which resembles the effect of anesthetic on their activity [66, 67]. Thus, exposure to anesthesia likely results in fast suppression of lacrimal gland function, consequent alteration of tear biochemistry, and development of DES symptoms. Consistently, SOD content in tears and intrinsic antioxidant activity of the tear fluid would be highly sensitive to protective action of SkQ1 on tear-producing glands. On the other hand, GPx and GR are predominantly intracellular proteins [68, 69] and their alterations in tears as a result of antioxidant premedication would be less noticeable, which agrees with our results. As for the cornea, the activities GPx and GR have been found in both corneal epithelium and endothelium [68, 69], and in our records, these enzymes become specifically upregulated within these cells under the SkQ1 action via a mechanism that is yet to be specified.

Apart from the improvement of tear antioxidant status, SkQ1 suppressed the development of proinflammatory reactions of the eye surface detectable in tears. In particular, the premedication averted completely acute elevation of TNF-*α* and delayed growth in IL-6 content, as well as promoted accumulation of anti-inflammatory IL-4 and IL-10 in tear fluid. Interestingly, the concentration of IL-10 increased more than 5-fold indicating a specific effect of SkQ1 on its expression/secretion. The revealed anti-inflammatory potential of SkQ1 agrees with its ability to prevent TNF-*α*-induced ICAM-1 overexpression in endothelium *in vitro* and *in vivo* and with its therapeutic effect in the case of various inflammatory disorders. Furthermore, similar antioxidant MitoQ mitigated symptoms of experimental colitis, which is governed, at least partially, by the prevention of IL-1*β* and IL-18 release [70]. In our case, anti-inflammatory effect may be associated with the upregulation of IL-10 by SkQ1. Indeed, overexpression of IL-10 was beneficial in treatment of ocular inflammatory diseases such as herpetic keratitis [71]. Furthermore, IL-10 is known to antagonize proinflammatory cytokines including TNF-*α* [72]. The latter is widely regarded as a master cytokine, downregulating of which would generally inhibit proinflammatory signaling [73].

The described increase in antioxidant and anti-inflammatory activity in the presence of SkQ1 agrees with our observations that premedication accelerates substantially tear film recovery. Given the fact that SkQ1 does not affect tear production, it can be suggested that the premedication generally improved biochemical properties of the tear film. Since the latter is produced by lacrimal and meibomian glands, as well as goblet cells of conjunctiva [65], it is possible that SkQ1 protects these tissues from the oxidative stress. Consistently, our preliminary results indicated positive effect of SkQ1 on clinical condition of conjunctiva of rabbits with anesthesia-associated DES (data not shown).

Taken together, our results demonstrate safety and efficacy of 7.5 *μ*M SkQ1 eye drop administration for prevention and treatment of DES. Such medication can be recommended for the patients and individuals at risk for the disease, including elderly people and those who were subjected to general anesthesia. In addition, our findings provide better understanding of pathophysiological mechanisms that are susceptible to antioxidant treatment in DES. These mechanisms include oxidative stress and inflammation that manifested in the cornea and tear film and resulted in apoptotic and necrotic changes in the corneal epithelium. SkQ1 and probably other mitochondria-targeted antioxidants can be used to suppress effectively these degenerative processes. Finally, we described novel qualities of SkQ1, such as its ability to upregulate antioxidant enzymes and anti-inflammatory cytokines. These data point to the feasibility of SkQ1 for treatment of various ocular surface pathologies different from DES, such as trauma, corneal erosions, and ulcers, as well as inflammatory disorders of the cornea and other eye tissues.

## Supplementary Material

Table S1. Parameters of the experimental groups.

## Figures and Tables

**Table 1:** Tear secretion in healthy rabbits medicated with SkQ1 eye drops.

SkQ1, *μ*M	Length, mm^∗^			
	Control		Medication	
	0 day	30 days	0 day	30 days
0.25	20.4 ± 0.92	22.11 ± 1.07	20.7 ± 1.18	23.2 ± 0.92 (*p* = 0.112^#^)
2.5	18.0 ± 1.4	17.0 ± 2.4	18.8 ± 0.9	21.4 ± 1.4 (*p* = 0.175^#^)
7.5	19.2 ± 0.4	18.4 ± 1.7	18.4 ± 2.1	19.1 ± 1.6 (*p* = 0.700^#^)

^∗^Length of the moistened Schirmer's test paper strip. ^#^Compared with the values measured prior to medication.

**Table 2:** Pathomorphological characteristics of the cornea in general anesthesia-induced DES.

Corneal compartment	Diagnosis	Parameter	Anesthesia time, h			
			Control	1	3	6
Epithelium	Superficial cell desquamation	Incidence, %	0	100	100	100
		MSS	0	2.3 ± 0.5^∗^	3.6 ± 0.6^∗^	4.3 ± 0.5^∗^
	Deep layer cell loss (erosion)	Incidence, %	0	0	100	100
		MSS	0	0	2.0 ± 0.9	4.3 ± 0.6
	Denudation	Incidence, %	0	0	66	100
		MSS	0	0	2.0 ± 1.5^∗^	4.0 ± 0.9^∗^
	Cell degeneration	Incidence, %	0	0	100	100
		MSS	0	0	3.3 ± 0.6^∗^	3.7 ± 0.5^∗^
	Signs of apoptosis	Incidence, %	0	0	33	100
		MSS	0	0	0.6 ± 0.9	3.6 ± 0.5^∗^
	Reepithelization	Incidence, %	0	0	0	0
		MSS	0	0	0	0
Stroma	Edema	Incidence, %	0	0	0	0
		MSS	0	0	0	0
	Inflammatory infiltration	Incidence, %	0	0	0	0
		MSS	0	0	0	0
	Neovascularization	Incidence, %	0	0	0	0
		MSS	0	0	0	0
Endothelium	Vacuolization	Incidence, %	0	0	0	0
		MSS	0	0	0	0
	Cell loss	Incidence, %	0	0	0	0
		MSS	0	0	0	0

Incidence and mean severity scores (MSS) were determined as described in Materials and Methods. ^∗^*p* < 0.05 compared with control.
