# Supplementary material for: Mitochondria-Targeted Antioxidant SkQ1 Prevents Anesthesia-Induced Dry Eye Syndrome
Source: Oxid Med Cell Longev. 2017 Oct 12;2017:9281519. doi: 10.1155/2017/9281519 (PMC5660788; doi:10.1155/2017/9281519)
Supplement: Supplementary file 1 — Table S1. Parameters of the experimental groups. [file 9281519.f1.docx]

| **Table S1. Parameters of the experimental groups.** | | | | |
| --- | --- | --- | --- | --- |
| Group | Anesthesia duration (h) | Premedication (µM SkQ1) | Treatment (µM SkQ1) | Analysis |
| 1(c)* | - | - | 0 (vehicle) | Visual examination, ophtalmoscopy, histology |
| 2 | - | - | 0.25 | Visual examination, ophtalmoscopy, histology |
| 3 | - | - | 2.5 | Visual examination, ophtalmoscopy, histology |
| 4 | - | - | 7.5 | Visual examination, ophtalmoscopy, histology |
| 5 | - | - | 25 | Visual examination, ophtalmoscopy, histology |
| 6(c) | - | - | 0 (vehicle) | Shirmer test |
| 7 | - | - | 0.25 | Shirmer test |
| 8 | - | - | 2.5 | Shirmer test |
| 9 | - | - | 7.5 | Shirmer test |
| 10(c) | 0 | - | - | Fluorescein test, histology, corneal biochemistry |
| 11 | 0.5 | - | - | Fluorescein test |
| 12 | 1 | - | - | Fluorescein test, histology, corneal biochemistry |
| 13 | 2 | - | - | Fluorescein test |
| 14 | 3 | - | - | Fluorescein test, histology, corneal biochemistry |
| 15 | 4 | - | - | Fluorescein test |
| 16 | 5 | - | - | Fluorescein test |
| 17 | 6 | - | - | Fluorescein test, histology, corneal biochemistry |
| 18(c) | 6 | 0 (vehicle) | - | Fluorescein test, histology, corneal biochemistry |
| 19 | 6 | 0.25 | - | Fluorescein test, histology |
| 20 | 6 | 2.5 | - | Fluorescein test, histology |
| 21 | 6 | 7.5 | - | Fluorescein test, histology, corneal biochemistry |
| 22(c) | 6 | - | 0 (vehicle) | Fluorescein test, histology |
| 23 | 6 | - | 0.25 | Fluorescein test |
| 24 | 6 | - | 2.5 | Fluorescein test |
| 25 | 6 | - | 7.5 | Fluorescein test, histology |
| 26(c) | 6 | 0 (vehicle) | - | Shirmer test, BUT test |
| 27 | 6 | 7.5 | - | Shirmer test, BUT test |
| 28(c) | 6 | 0 (vehicle) | - | Tear biochemistry |
| 29 | 6 | 7.5 | - | Tear biochemistry |
| *control group | | | | |
